# The differential susceptibility of spores from virulent and attenuated *Bacillus anthracis* strains to aldehyde- and hypochlorite-based disinfectants

**DOI:** 10.1002/mbo3.45

**Published:** 2012-10-11

**Authors:** Jordon K March, Marissa N Cohen, James M Lindsey, D A Millar, Chinn-Woan Lowe, Annette J Bunnell, Kim L O'Neill, G Bruce Schaalje, Richard A Robison

**Affiliations:** 1 Department of Microbiology and Molecular Biology, 775 WIDB, Brigham Young UniversityProvo, Utah, 84602; 2 Department of Statistics, 230 TMCB, Brigham Young UniversityProvo, Utah, 84602

**Keywords:** Anthrax, *Bacillus anthracis*, disinfection, inactivation kinetics, spore, sporicide

## Abstract

This study compared the sensitivity of spores from virulent and attenuated *Bacillus anthracis* strains in suspension to inactivation by various chemical disinfectants. Spore suspensions from two virulent strains (A0256 and A0372) and two attenuated strains (Sterne and A0141) of *B. anthracis* were tested against two aldehyde-based disinfectants and one hypochlorite-based disinfectant. A novel statistical model was used to estimate 4-log_10_ reduction times for each disinfectant/strain combination. Reduction times were compared statistically using approximate *Z* and χ^2^ tests. Although there was no consistent correlation between virulence and increased sporicidal resistance across all three disinfectants, spores from the two virulent and two attenuated strains did display significantly different susceptibilities to different disinfectants. Significant disinfectant–strain interactions were observed for two of the three disinfectants evaluated. The comparative results suggest that the use of surrogate organisms to model the inactivation kinetics of virulent *B. anthracis* spores may be misleading. The accuracy of such extrapolations is disinfectant dependent and must be used with caution.

## Introduction

The intentional release of *Bacillus anthracis* spores through the U.S. Postal Service has generated significant interest in chemical disinfectants that are capable of inactivating spores from virulent *B. anthracis* strains. However, government regulations and safety concerns prevent many laboratories from using virulent *B. anthracis* spores in disinfection studies. Biosafety Level 3 (BSL-3) operating conditions are required when working with high concentrations of virulent *B. anthracis* spores, whereas the use of surrogate organisms, such as *B. anthracis* Sterne (an attenuated strain), is much less stringently regulated. Currently, manufacturers seeking to validate their products against virulent *B. anthracis* spores often use attenuated strains for efficacy testing, with the assumption that spores from attenuated and virulent strains are equally susceptible to various sporicidal agents.

Virulent strains of *B. anthracis* contain a lone circular chromosome and two large virulence plasmids, pXO1 and pXO2 (Keim et al. 2009). The pXO1 plasmid possesses the *lef* (lethal factor), *cyaA* (edema factor), *pagA* (protective antigen), *atxA* (anthrax toxin activator), and *bslA* (adhesion protein) genes (Dai et al. 1995; Kern and Schneewind 2008). The pX02 plasmid carries a series of *cap* genes which encode proteins involved in the biosynthesis of the poly-d-glutamate capsule (Dai et al. 1995). The functions and regulatory mechanisms of many other genes on the virulence plasmids are still unknown. Attenuated strains are characterized by the absence of one or both virulence plasmids. For example, the *B. anthracis* Sterne strain harbors pXO1, but is missing pXO2. It is unclear whether genes encoded on these plasmids could affect spore resistance to certain disinfectants.

Despite the known physical and genetic differences between virulent and attenuated strains of *B. anthracis*, there are relatively few published studies that compare the spore susceptibilities of these strains to various disinfectants. The few studies that address this topic have reported conflicting results. Sagripanti et al. (2007) investigated the susceptibility of spores from virulent *B. anthracis* strains deposited on various surfaces (metal, glass, and rubber) exposed to a range of disinfectants. The susceptibility of these spores was compared with that of spores from attenuated *B. anthracis* strains, as well as to spores from other surrogate organisms, under identical experimental conditions. The study reported similar sensitivities for all *Bacillus* strains and species tested. However, a more recent study reported differential sensitivities between virulent *B. anthracis* spores and spores from surrogate organisms, including attenuated *B. anthracis* strains, when deposited on a metal surface and exposed to different disinfectants (Majcher et al. 2008).

The effect of chlorine treatment on spores in suspension has also provided some comparative information regarding the sensitivities of spores from attenuated and virulent *B. anthracis* strains. Initially, it was reported that spores from the *B. anthracis* Ames (virulent) strain in suspension were slightly less susceptible to inactivation by chlorine than spores from the *B. anthracis* Sterne strain (Rose et al. 2005). Upon further investigation, a substantial difference between the sensitivities of *B. anthracis* Ames spores and spores of *B. anthracis* Sterne was observed, with the virulent Ames spores being much more resistant to chlorine while in suspension (Rice et al. 2005).

Preliminary studies in our laboratory have also indicated variable susceptibilities to chemical disinfectants among spores from virulent and attenuated *B. anthracis* strains. To further investigate these observations, we tested three high-level disinfectants against spores from two virulent (A0256 and A0372) and two attenuated (Sterne and A0141) *B. anthracis* strains. The following high-level disinfectants were used: alkaline glutaraldehyde (GTA), *ortho*-phthalaldehyde (OPA), and stabilized sodium hypochlorite (SSH).

GTA (pentanedial; CHO(CH_2_)_3_CHO) is an effective sporicide that displays optimal antimicrobial activity under basic conditions (Power and Russell 1990; Russell 1990; Coates 1996; Angelillo et al. 1998; Tennen et al. 2000). Consequently, aqueous GTA solutions are activated by the addition of alkalinizing agents. Once activated, these solutions tend to rapidly polymerize, which decreases the concentration of free dialdehyde and consequently the antimicrobial activity (Kelsey et al. 1974; Gorman et al. 1980; Russell 1990). Accordingly, manufacturers recommend that GTA solutions be discarded 2–3 weeks after activation (Gorman et al. 1980). GTA inactivates spores by cross-linking outer proteins and blocking normal germination events (Tennen et al. 2000). GTA was investigated in this study because it has been available commercially for many years and is a frequently used high-level disinfectant and cold sterilant (Lane et al. 1966).

In October 1999, the Food and Drug Administration cleared OPA (C_6_H_4_(CHO)_2_) for use as a high-level disinfectant (Rutala and Weber 2001). OPA has demonstrated exceptional bactericidal and mycobactericidal activity (Gregory et al. 1999; Walsh et al. 1999a). However, it has also proven to be less effective against bacterial endospores than GTA (Walsh et al. 1999a). Although its mechanism of action has not been thoroughly investigated, the results from several studies indicate that OPA acts in a manner similar to GTA (McDonnell and Russell 1999; Walsh et al. 1999b). However, the degree to which OPA cross-links outer proteins appears to be much less extensive than GTA (Walsh et al. 1999b). In addition, OPA does not rapidly polymerize, which significantly increases its stability in comparison with GTA (Rutala and Weber 2001). OPA was included in this study because it is frequently used in healthcare facilities and has relatively low sporicidal activity.

Hypochlorite-based disinfectants, like SSH, are effective against a wide-variety of microorganisms such as viruses, vegetative bacterial cells, and bacterial endospores (McDonnell and Russell 1999). Hypochlorite inactivates spores through the oxidization of fatty acids and proteins (Young and Setlow 2003). At sublethal concentrations, hypochlorite significantly damages proteins in the spore coat (McDonnell and Russell 1999; Young and Setlow 2003). At higher concentrations, the spore coat will actually separate from the cortex, resulting in spore lysis (Kulikovsky et al. 1975). Hypochlorite also damages the inner membrane, destroying germinant receptors and cortex lytic enzymes, thus inhibiting the process of germination (Young and Setlow 2003). SSH remains stable for 2 years from date of manufacture. It also contains a detergent and an anticorrosive ingredient that makes it less damaging to surfaces or equipment than conventional hypochlorite solutions. SSH was included in this study because of its frequent use in healthcare settings and its known sporicidal properties.

In this study, we compared the inactivation kinetics of virulent *B. anthracis* spores with those of attenuated *B. anthracis* spores upon exposure to the three common high-level disinfectants that were described above. This allowed us to evaluate the hypothesis that spores from virulent and attenuated *B. anthracis* strains are equally susceptible to inactivation by various disinfectants.

## Materials and Methods

### Disinfectants

Test solutions of GTA and OPA were prepared by the manufacturer (Advanced Sterilization Products, Irvine, CA). Solution preparation included activation, followed by dilution to the minimum effective concentration (MEC). The MEC is the minimum concentration specified by the manufacturer that will perform according to stated label claims. Chemical titrations confirmed that GTA and OPA solutions were at their respective MECs. Due to its tendency to rapidly polymerize, the GTA solutions were tested within 24 h of MEC verification. Alternatively, the OPA solutions were tested within a week of MEC verification, because rapid polymerization was not a concern. The GTA and OPA solutions were tested at an MEC of 1.5% and 0.3%, respectively. The SSH solution consisted of 0.55% stabilized sodium hypochlorite and a detergent. This agent was tested undiluted as recommended by the manufacturer (Dispatch, Caltech Industries Inc, Midland, MI).

### Neutralizing agents

Controls were conducted to ensure adequate neutralization of the disinfectants by combining 100 μL of a spore suspension (containing approximately 1 × 10^4^ spores mL^−1^) with 1 mL of disinfectant and 9 mL of the appropriate neutralizer. A 1% (w/v) glycine solution, prepared just prior to use, was used to neutralize the two aldehyde-based disinfectants. The sodium hypochlorite-based disinfectant was inactivated using a freshly prepared 1.4% (w/v) sodium thiosulfate solution. The neutralized solution (containing approximately 100 spores mL^−1^) was allowed to rest for 20 min before being assayed for the number of viable spores. Neutralizer controls were plated in triplicate using a membrane filtration system (E-Z Pak 0.45 μm, Millipore Corporation, Billerica, MA).

### *Bacillus* strains

Strains of *B. anthracis* used in this study are shown in Table 1. Strains were confirmed as *B. anthracis* by the gas chromatographic analysis of cellular fatty acids using an Agilent 6890 Series Gas Chromatograph (Santa Clara, CA) and software purchased from MIDI Inc (Newark, DE). Real-time polymerase chain reaction (PCR) assays targeting chromosomal and plasmid gene sequences (Tetracore Inc, Rockville, MD) were used to definitively identify the strains as *B. anthracis* and to confirm the plasmid composition of each strain.

**Table 1:** *Bacillus anthracis* strains used in this study

Strain denomination		Plasmids^1^			
			
Name	Alternate designation	pXO1	pXO2	Pathogenesis	Source
A0256	K6428/11	+	+	Virulent	Louisiana State University
A0372	K0404/#40/BA1000 Vollum	+	+	Virulent	Louisiana State University
Sterne	Sterne 1043	+	−	Attenuated	Los Alamos National Laboratory
A0141	Pasteur	−	+	Attenuated	Louisiana State University

1 Plasmids are indicated as present (+) or absent (−).

### Spore suspension preparation

Spore suspensions of all *B. anthracis* isolates were prepared under BSL-3 operating conditions. Aliquots of a saturated culture (100 μL) were spread over the surface of plates containing a modified version of Tarr's sporulation agar. The medium was formulated as described by Dragon and Rennie (2001), with the addition of 12-g nutrient broth (Becton, Dickson and Company, Sparks, MD). Plates were incubated at 37°C for 7–14 days, or until cultures exhibited >95% refractile spores. The percent of refractile spores was monitored on a daily basis by phase-contrast microscopy. Spores were harvested and suspended in 10 mL of ice-cold sterile HPLC water before being heat shocked in an 80°C water bath for 10–15 min to kill vegetative cells. To select for resistant spores, aliquots of the heat-shocked spore suspension (100 μL) were spread over modified Tarr's sporulation agar plates using rolling 4-mm glass beads until complete surface inoculation was achieved. Plates were again incubated at 37°C and monitored daily by phase-contrast microscopy until cultures exhibited >95% refractile spores. Spores were harvested and suspended in ice-cold water containing 0.05% Tween 20 (WT20) before being heat shocked in a 60°C water bath for 30 min to kill any remaining vegetative cells. The spore suspension was pelleted by centrifugation at 5000*g* for 15 min at 4°C and suspended in ice-cold WT20. After incubating for 16–18 h at 4°C to promote the lysis of dead vegetative cells, the spore suspension was pelleted, as described above, and suspended in ice-cold WT20. The spore suspension was further purified by adding 95% ethanol until a final ethanol concentration of 75% was achieved. The spores in the ethanol suspension were incubated for 20 min at room temperature prior to being washed in ice-cold WT20. After centrifugation, the resulting pellet was suspended in 5-mL ice-cold WT20. The spore suspension was quantified using serial dilution and triplicate plating using a membrane filtration system. All spore suspensions were stored at 4°C. Spore suspensions for each *B. anthracis* strain were prepared in an identical manner.

### Evaluation of disinfectant efficacy

A spore suspension (containing approximately 1 × 10^6^ spores mL^−1^) was vortexed for 2–3 min to ensure a homogenous mixture. Aliquots of the spore suspension (1 mL) were transferred to vials containing 9 mL of the chosen disinfectant (previously equilibrated to 20°C) at time zero. The vial was vortexed for 30 sec and placed back in a 20°C water bath.

Samples of the spore/disinfectant suspension were taken after various exposure times. The vial was removed from the water bath and vortexed for 30 sec before a 1-mL aliquot of the solution was removed to 9 mL of an appropriate neutralizing agent. The spore/neutralizer suspension was vortexed for 30 sec and allowed to stand for 20 min to allow complete neutralization of the active ingredient. The neutralized solution was serially diluted in physiological saline solution (PSS), and the viable spores of 1-mL aliquots from each dilution were quantified in triplicate using membrane filtration. Filter membranes were incubated on plates containing Columbia agar (Becton, Dickson and Company, Sparks, MD) at 37°C. Colonies were counted after incubating for 18, 24, and 36 h. If new germination continued beyond 36 h, the plates were counted at regular intervals until increases in colony numbers were no longer observed. This occurred most frequently with spores exposed to OPA. Assays were repeated in triplicate for each disinfectant/strain combination.

### Statistical methods

#### Experimental design

To determine differences in spore susceptibilities, three disinfectants were tested against spores from four strains of *B. anthracis* (two virulent and two attenuated). On each test day, a different combination of strain and disinfectant was selected at random until each combination was repeated three times. Each dilution assayed for viable spores was plated in triplicate, and these counts were averaged to obtain the estimate for each dilution. A novel statistical model was then used to determine a 4-log_10_ reduction time for each combination of strain and disinfectant.

The statistical model was based on the standard Hom model for describing the kinetics of microbial disinfection (Hom 1972). The Hom model allowed for the tailing behavior that results from a small number of spores that appear to be very resistant to the effects of the disinfectant. However, the Hom model did not allow for any shouldering behavior, a time lag where apparently little to no inactivation occurs. Several studies have extended the Hom model to allow for shouldering due to disinfectant decay (Haas and Joffe 1994; Lambert 2001). In the disinfection of bacterial endospores, however, the shouldering behavior most likely results from the physical properties of the endospores which slow disinfectant penetration (Broadwater et al. 1973; Fernando and Othman 2006). In this study, the Hom model was modified to allow for (i) the shouldering behavior due to the physical properties of spores, (ii) Poisson errors associated with the sampling and counting of spores, (iii) hierarchical parameter deviations associated with specific tubes, and (iv) the effects of serial dilutions on spore counts. The statistical model is described in detail below.

#### Analysis of data

The method of maximum likelihood was used to fit the following nonlinear hierarchical model to counts (*y*_ijkl_) of viable spores at specified dilutions (*dil*_ijkl_) from the various tubes (*k*) at specified times (*tim*_ijkl_) following application of the disinfectants (*i*) to the spores of various *B. anthracis* strains (*j*):


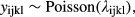



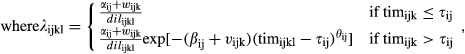


and 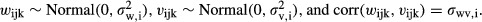


There are four parameters in the model for the mean count for every disinfectant/strain combination, *α*_ij_, *β*_ij_, *θ*_ij_, and *τ*_ij_. The *α*_ij_ parameters represent initial counts. The *β*_ij_ parameters are disinfection rate constants. The *θ*_ij_ parameters allow the disinfection patterns to exhibit tailing behavior. The parameters *τ*_ij_ are necessary because of the very sharp initial shoulder in the disinfection patterns. They represent the lag times or times during which apparently little to no killing takes place. The model was fitted to the data using the NLMIXED (nonlinear mixed model) procedure of the SAS® software (SAS Institute Inc, Cary, NC).

Four-log_10_ reduction times were estimated for each disinfectant/strain combination as


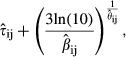


and approximate standard errors were calculated using the multivariate delta method. Four-log_10_ reduction times were compared statistically among strains and disinfectant using approximate *Z* and χ^2^ tests.

Four-log_10_ reduction times are frequently used in disinfection studies (Kozma-Sipos et al. 2010; Oliveira et al. 2010; Cho et al. 2011; Prasad et al. 2011). In this study, the 4-log_10_ reduction times were compared because higher estimates would necessitate extrapolation beyond the data generated. This stems from the fact that each spore suspension had an original concentration of approximately 1 × 10^6^ spores mL^−1^. Then, through exposure to the disinfectant and subsequent neutralizer, the concentration underwent a 1:100 dilution, thus giving a maximum observable concentration of 1 × 10^4^ spores mL^−1^. Therefore, a 4-log reduction time was the maximum kill that could be detected.

## Results and Discussion

Viable spore counts for each disinfectant/strain combination were obtained and analyzed. The approximate 4-log_10_ reduction times and standard errors for each combination are shown in Table 2. When tested against the GTA solution, the 4-log_10_ reduction time for the virulent strain A0256 was significantly greater than the virulent strain A0372 and both of the attenuated strains (Sterne 1043 and A0141). Alternatively, when tested against the OPA solution, the virulent A0256 strain and the Sterne strain exhibited 4-log_10_ reduction times that were not significantly different from one another, but were significantly longer than the other two strains. All of the strains tested showed very similar 4-log_10_ reduction times when tested against the SSH solution; differences in the 4-log_10_ reduction times were not significant in this case.

**Table 2:** Estimated 4-log_10_ reduction times and standard errors for spores from four different *Bacillus anthracis* strains using three different disinfectants

		4 log_10_ reduction times (min)			Virulence plasmids	
			
Disinfectant	Strain	Estimate^1^		SE	pXO1	pXO2
GTA	A0256	34.46	a	3.52	+	+
	A0372	20.13	b	1.97	+	+
	Sterne	19.22	b	5.54	+	−
	A0141	19.99	b	1.01	−	+
OPA	A0256	157.51	a	4.46	+	+
	A0372	113.49	b	14.48	+	+
	Sterne	150.96	a	5.65	+	−
	A0141	109.24	b	2.79	−	+
SSH	A0256	20.02	a	0.60	+	+
	A0372	19.91	a	0.62	+	+
	Sterne	22.70	a	1.61	+	−
	A0141	23.28	a	3.89	−	+

GTA, 1.5% alkaline glutaraldehyde; OPA, 0.3% *ortho*-phthalaldehyde; SSH, 0.55% stabilized sodium hypochlorite.

1 Estimates for *Bacillus anthracis* strains (within disinfectants) followed by the same letter are not significantly different (*P* > 0.05).

Statistical test results for interactions between bacterial strains and disinfectants are displayed in Table 3. The disinfectant–strain interaction was highly significant when all the disinfectants were analyzed together and when the GTA and SSH solutions were analyzed together. The results also indicated that each *B. anthracis* strain responded differently to the GTA and OPA. However, the SSH affected all of the *B. anthracis* strains in a similar manner.

**Table 3:** Significance of interactions between *Bacillus anthracis* strains and disinfectants

	4-log reduction times (min), χ^2^ (degrees of freedom)	Significance^1^
Disinfectant–strain interaction		
GTA/OPA/SSH	96.69 (6)	Yes
GTA/SSH	15.46 (3)	Yes
Differences among strains		
GTA	15.85 (3)	Yes
OPA	106.17 (3)	Yes
SSH	3.35 (3)	No

GTA, 1.5% alkaline glutaraldehyde; OPA, 0.3% *ortho*-phthalaldehyde; SSH, 0.55% stabilized sodium hypochlorite.

1 *P* < 0.05.

In general, these results show that there is not a consistent response among spores from virulent and attenuated strains of *B. anthracis,* across the disinfectants tested. Rather, the data showed a significant disinfectant–strain interaction. These differences were unpredictable and varied with the type of disinfectant evaluated. These results also indicated that virulence per se is not the only factor involved in spore susceptibility. Disinfectant susceptibilities may, in part, be influenced by the presence or absence of the virulence plasmids as well as possible variations in chromosomal genes that can occur between strains. In the three cases where some strains were significantly more resistant than others, pXO1 was present in every instance, whereas pXO2 was only present in two of the three cases (Table 2).

Experiments involving the inactivation of microorganisms by chemical disinfectants commonly present results using a linear logarithmic decay curve. However, data from this study showed that spore inactivation does not always occur in a strict log-linear fashion, but rather exhibits an initial shoulder and a late tail. The nonlinear statistical model described here takes these shouldering and tailing tendencies into account, giving a more accurate representation of the inactivation kinetics.

**Figure 1:**
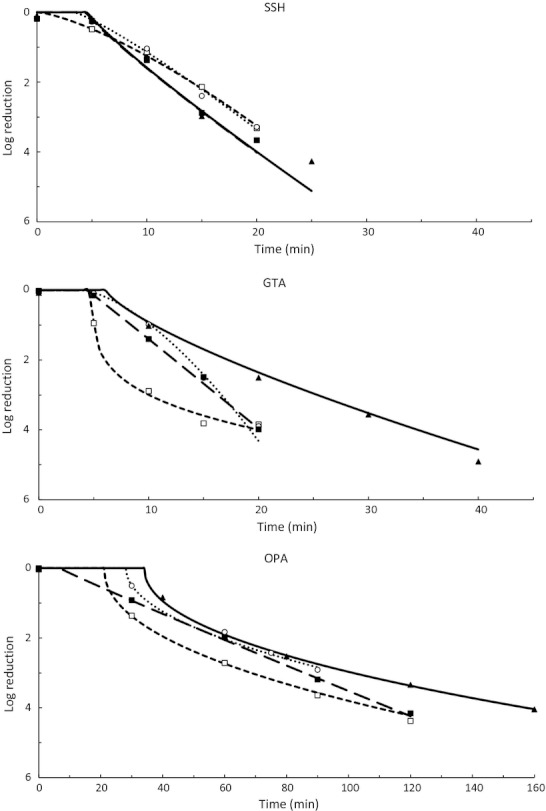
Inactivation kinetics of *Bacillus anthracis* spores from virulent strains A0256 (▲,**―**) and A0372 (▪,**– –**) and attenuated strains Sterne (o, **····**) and A0141 (□, **—**) upon treatment with 1.5% alkaline glutaraldehyde (GTA), 0.3% *ortho*-phthalaldehyde (OPA), and 0.55% stabilized sodium hypochlorite (SSH).

Figure 1 shows the nonlinear model described above fitted to the disinfectant efficacy test data. Data from both the GTA and OPA solutions exhibited prominent shouldering and tailing behavior, indicating that a log-linear model would introduce error if applied to these data. The data from the SSH solution appeared to be more linear and did not exhibit as pronounced shouldering or tailing behavior as the aldehyde-based disinfectants.

The differences in spore resistance may be influenced by many factors including, but not limited to, the presence or absence of virulence plasmids, chromosomal-based genetic differences between strains, and by interactions between gene products of the plasmids and the chromosome. Further research is needed to more specifically determine the reasons for the highly significant disinfectant–strain interactions seen in this study. Testing with a larger number of isolates and a wider range of disinfectants may help define the significant variables involved. For this reason, the use of data from surrogate organisms to model inactivation kinetics of virulent *B. anthracis* strains may be misleading, and caution should be used when extrapolating sporicidal results from attenuated strains to virulent strains, depending on the disinfectant used.
